# Association of Eumycetoma and Schistosomiasis

**DOI:** 10.1371/journal.pntd.0002241

**Published:** 2013-05-23

**Authors:** Jaap J. van Hellemond, Alieke G. Vonk, Corné de Vogel, Rob Koelewijn, Norbert Vaessen, Ahmed H. Fahal, Alex van Belkum, Wendy W. J. van de Sande

**Affiliations:** 1 Erasmus MC, Department of Medical Microbiology and Infectious Diseases, Rotterdam, The Netherlands; 2 Harbour Hospital and Institute for Tropical Diseases, Laboratory for Parasitology, Rotterdam, The Netherlands; 3 Mycetoma Research Centre, University of Khartoum, Khartoum, Sudan; 4 bioMerieux, Microbiology R&D, La Balme Les Grottes, France; Public Health Intitution of Turkey, Turkey

## Abstract

Eumycetoma is a morbid chronic granulomatous subcutaneous fungal disease. Despite high environmental exposure to this fungus in certain regions of the world, only few develop eumycetoma for yet unknown reasons. Animal studies suggest that co-infections skewing the immune system to a Th2-type response enhance eumycetoma susceptibility. Since chronic schistosomiasis results in a strong Th2-type response and since endemic areas for eumycetoma and schistosomiasis do regionally overlap, we performed a serological case-control study to identify an association between eumycetoma and schistosomiasis. Compared to endemic controls, eumycetoma patients were significantly more often sero-positive for schistosomiasis (p = 0.03; odds ratio 3.2, 95% CI 1.18–8.46), but not for toxoplasmosis, an infection inducing a Th1-type response (p = 0.6; odds ratio 1.5, 95% CI 0.58–3.83). Here, we show that schistosomiasis is correlated to susceptibility for a fungal disease for the first time.

## Author Summary

Eumycetoma is a mutilating fungal disease of mainly the foot and is found in (sub)tropical regions such as Sudan. At the moment it is not understood why some people develop eumycetoma and others not. In the regions were eumycetoma is prevalent many other infections are also found. These infections could alter the immune system which makes people more or less susceptible in obtaining another infection. One of the infections with such an effect is Schistosomiasis. In Africa, eumycetoma is found in regions were schistosomiasis is prevalent. In this study we show that eumycetoma patients more often have antibodies against *Schistosoma* species, than healthy controls from the same region. In contrast, eumycetoma patients did not have more often antibodies against *Toxoplasma* species. This might implicate that schistosomiasis predisposes eumycetoma development. If schistosomiasis indeed predisposes eumycetoma development, eradicating *Schistosoma* in a population could also lower the number of eumycetoma cases in that area, which in the end could lead to intervention strategies not only for schistosomiasis but also for eumycetoma.

## Introduction

**Figure 1:**
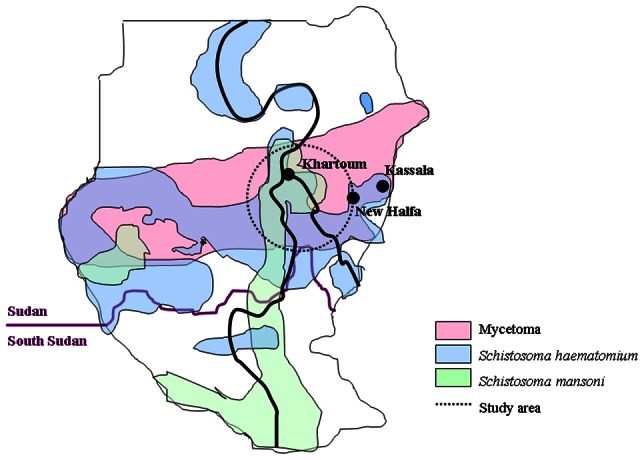
Endemic regions of mycetoma, *Schistosoma haematomium* and *Schistosoma mansoni* in Sudan. The area from which our sera were collected is encircled. The cities of Khartoum, New Halfa and Kassala are indicated.

Eumycetoma is a chronic granulomatous subcutaneous infectious disease endemic in many tropical and sub-tropical regions in the so-called mycetoma belt between 30°N and 15°S of the equator [1]. Sudan is a country with the highest country-wide prevalence of eumycetoma (Figure 1). In a recent survey conducted by the Mycetoma Research Centre, it appeared that in the endemic villages in the Gezira area of Sudan 2% of the population has eumycetoma (Prof. A. Fahal, personal communication) [2], [3]. Although mycetoma can be caused by a variety of bacterial and fungal micro-organisms, most mycetoma cases in Sudan (ca. 70%) are caused by the fungus *Madurella mycetomatis* (eumycetoma) [4]. Based on antibody measurements in earlier studies it was noted that although most people living in endemic areas in the Sudan have developed antibodies against *M. mycetomatis*, and thus have been exposed to *M. mycetomatis*, only few of them actually developed eumycetoma [5], [6]. To date, it is unknown why some people are predisposed to develop eumycetoma.

Multiple explanations can be considered for the scanty susceptibility to eumycetoma. Firstly, genetic differences in the pathogen might exist that could lead to pathogenic and non-pathogenic variants of *M. mycetomatis*. Secondly, genetic polymorphisms in the host involved in sex hormone synthesis and neutrophil function have already been associated with eumycetoma, indicating that the genetic make-up of the host is a crucial factor in susceptibility to eumycetoma development [7]–[9]. Furthermore, a combination of specific genetic requirements and capabilities in both the pathogen and the host could lead to an even more sporadic development of eumycetoma. Thirdly, temporal conditions influencing the host immune response, such as co-infections, nutritional status, use of antibiotics and/or immune suppression or skewing may also play a role in susceptibility to *M. mycetomatis*. This possibility is supported by the observation that *M. mycetomatis* could only induce eumycetoma in animals in the presence of an adjuvant predisposing towards a Th2-response [10] but not a Th1-response [11], [12].

Skewing of the immune response is highly affected by invasive pathogens [13], and therefore, co-infections could play a critical role in eumycetoma [14]. In this respect, infections inducing a strong and long-lasting Th2-type of immune response could favour the development of eumycetoma disease most. Schistosomiasis seems to meet such requirements for the following reasons. Firstly, schistosomiasis induces a long-lasting Th2-type immune response that is strong enough to even convert an already established Th1-response [15], [16]. Secondly, in endemic countries schistosomiasis is often a chronic life-long disease. Even when patients are regularly treated for schistosomiasis, their continuous exposure to the parasite during fresh water contacts and the lack of the development of immunity against schistosomes will rapidly result in a re-infection with a persistent Th2-response.

Based upon the above mentioned observations, and the fact that we recently have shown that eumycetoma patients have increased concentrations of circulating IL-10 [7], we hypothesize that schistosomiasis, which induces a Th2-type response with elevated levels of IL-10, might increase the susceptibility to eumycetoma, whereas toxoplasmosis which induces a Th1-type response [16], [17], should not be associated with eumycetoma.

## Methods

### Study population

A total of 84 serum samples was taken from 53 eumycetoma patients and 31 controls, matched for age and gender, in the endemic areas of Sudan between 2001 and 2008 (Table 1). Serum samples were stored at −80°C until assay. The patients' demographic characteristics were recorded and that included gender, duration of disease, lesion size and site of infection. Eumycetoma was confirmed by culture and molecular identification based on sequencing the Internal Transcribed Spacer [18]. Written informed consent was obtained from all participants and ethical clearance was obtained from Soba University Hospital Ethical Committee, Khartoum, Sudan.

**Table 1:** Details of the study population.

		Eumycetoma patients	Healthy endemic controls	p-value
**Number (n)**		53	31	
**Gender (male/female)**		51/2	30/1	p = 1.00**
**Mean age years (range)**		25.8 (13–74)	28.1 (20–47)	p = 0.30***
**Eumycetoma duration in years (range)**		6.3 (1–15)	NA*	
**Lesion site**		Foot	NA	
**Lesion size (n)**	Large	18	NA	
	Medium	19	NA	
	Small	16	NA	
**Causative agent**		*M. mycetomatis*	NA	
**Schistosoma serology** ****	Positive (n)	30	9	p = 0.03**
	Negative (n)	19	18	
**Toxoplasma serology**	Positive (n)	24	11	p = 0.5**
	Negative (n)	29	20	

* NA: not applicable.

** Chi square.

*** Mann-Whitney.

**** 4 patients and 4 controls had an equivocal outcome after multiple testing, they were left out of the analysis.

### Serological response towards antigens of *Toxoplasma*, *Schistosoma* and *Madurella* spp

Specific IgG antibodies against *Toxoplasma gondii* were determined with the commercially available Toxo IgG II assay on the automated Liaison serology platform according to the manufacturer's protocol (Diasorin, Saluggia, Italy). Antibody levels against *Schistosoma* species were determined as described before by a combination of a commercial indirect hemagglutination test with *Schistosoma mansoni* adult worm antigens (IHA; Fumouze Laboratories, Levallois-Perret Cedex, France) and an enzyme-linked immunosorbent assay with homemade *S. mansoni* Soluble Egg Antigens (SEA) [19]. The IHA was considered positive when the titre was ≥1∶80 and the SEA ELISA was considered positive when the Optical Density (O.D.) at 492 nm was ≥0.15. For optimal specificity, *Schistosoma* spp. serology was only considered positive when a positive result was obtained in both the IHA and SEA-ELISA tests.

Antibody levels against *Madurella mycetomatis* Translationally Controlled Tumour Protein (TCTP) were measured with Luminex Technology as described before [5].

### Statistical analysis

Difference in positive and negative serology for schistosomiasis and toxoplasmosis between eumycetoma patients and endemic controls were calculated with the Chi square test (GraphPad Instat 3.00) by determining both the two-sided p-value and the Odds Ratio using Yates correction. The 95% confidence interval of the Odds Ratio was calculated using the approximation of Woolf. The Mann-Whitney *U* test was used to compare differences between IgG levels raised against the MmTCTP antigen in the study populations (GraphPad Instat 3.00). The Kruskal-Wallis test (SPSS Inc 17) was used to test if concentrations of antibodies against *Schistosoma* spp. differed significantly between patients with larger lesions compared to patients with smaller lesions, by including size (small, moderate, large) as the grouping variable. A value of p<0.05 was considered significant.

## Results

Between 2001 and 2008, 53 patients and 31 endemic controls were included in the study. Most patients were male and had eumycetoma of the foot (Table 1). All patients and controls came from the same area mainly from Central Sudan as indicated in Figure 1.

**Figure 2:**
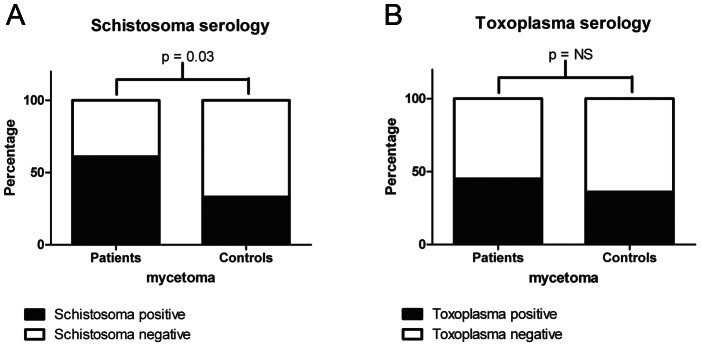
Percentage of eumycetoma patients and matched controls with positive serology for schistosomiasis and toxoplasmosis. Percentage of eumycetoma patients and matched controls with positive serology for schistosomiasis (panel A) and toxoplasmosis (panel B). Eumycetoma patients were significantly more often sero-positive for schistosomiasis when compared to matched controls (p<0.05; Chi square test).

As shown in Figure 2, eumycetoma patients were significantly more often sero-positive for *Schistosoma* infections as compared to endemic controls (Chi square, p = 0.03). In other words, an association exists between the infections caused by *Madurella mycetomatis* and *Schistosoma* spp. since the odds ratio for co-occurring schistosomiasis in eumycetoma patients is 3.2 (95% Confidence Interval 1.18–8.46). In contrast, eumycetoma patients were not significantly more often sero-positive for *Toxoplasma gondii* infections (Figure 2, Chi square, p = 0.5) thus the risk for developing eumycetoma does not seem to be increased in case of concurrent toxoplasmosis (Odds ratio 1.5, 95% Confidence Interval: 0.60–3.75). No correlation was found between sero-positivity for schistosomiasis and sero-positivity for toxoplasmosis (Chi square, p = 0.1, Odds ratio 0.44, 95% Confidence Interval: 0.14–1.32).

**Figure 3:**
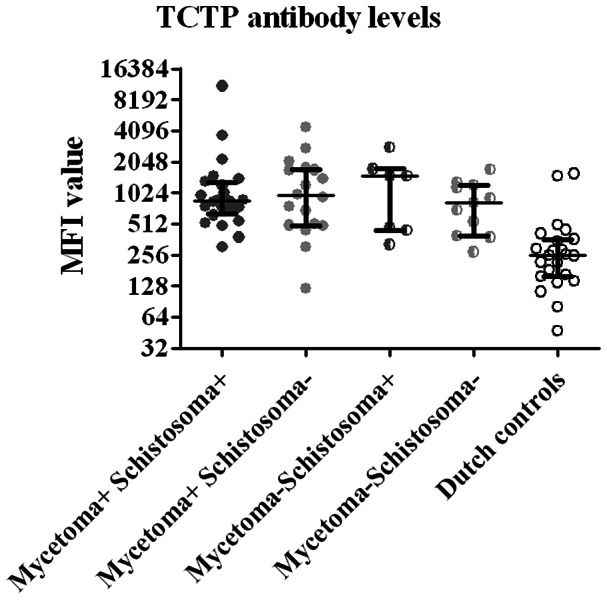
Median fluorescence intensity of *Madurella mycetomatis* TCTP in various groups. Median fluorescence intensity (MFI) values reflecting levels of antigen-specific IgG for recombinant *Madurella mycetomatis* his-tagged TCTP in eumycetoma patients with positive *Schistosoma*-serology (mycetoma+ *Schistosoma*+), eumycetoma patients without positive *Schistosoma*-serology (mycetoma+ *Schistosoma*−), healthy endemic controls with positive *Schistosoma*-serology (mycetoma− *Schistosoma*+), healthy endemic controls without positive *Schistosoma*-serology (mycetoma− *Schistosoma*−) and Dutch controls. Each symbol represents a single patient or control. Horizontal Lines indicate median levels of anti-*Madurella* antibodies. Significance was calculated with the Mann-Whitney test. Only antibody levels measured in Dutch controls were significantly lower than any other group (all p<0.01).

Positive serology for either schistosomiasis or toxoplasmosis was not correlated to the size of the eumycetoma lesion (Chi square, p = 0.8 and p = 0.2, respectively, data not shown). Since schistosomiasis is known to reduce the humoral immune response against co-infecting pathogens [20], [21], we investigated the antibody response against *M. mycetomatis* antigen TCTP. The antibody levels against TCTP did not differ between the eumycetoma patients with positive schistosomiasis serology and those with negative schistosomiasis serology, nor did they differ between matched endemic controls with positive schistosomiasis serology and healthy endemic controls with negative schistosomiasis serology (Figure 3). This suggests that schistosomiasis does not influence the humoral immune response against the TCTP antigen of *M. mycetomatis*.

## Discussion

Schistosomiasis is a chronic disease with an estimated 200 million people infected in subtropical countries [22]. Therefore, almost all schistosomiasis patients will subsequently be infected by one or more additional pathogens. Although a prior infection with schistosomes often has an effect on the subsequent infection by a virus, bacterium, protozoan or other helminth, schistosomiasis can cause both an increase or a decrease in the severity of the subsequent infection for yet unknown reasons (reviewed in Abruzzi and Fried [23]. Decreased subsequent disease severity was observed for co-infections with *Helicobacter pylori*, *Fasciola hepatica*, *Echinostoma* and with *Plasmodium* in case of *S. haematobium* schistosomiasis [23]. In addition, a worsened outcome of a subsequent co-infection has been described for HIV (reduced viral clearance) [24], *Leishmania donovani* [25], *Toxoplasma gondii*, *Entamoeba histolytica* infections and *Plasmodium* in case of *S. mansoni* schistosomiasis [23], [26]. The effect of subsequent fungal infections has not been addressed yet.

Since eumycetoma infections can only be established in animals with adjuvants inducing a strong Th2-type immune response, we hypothesized that co-infections inducing a Th2-type immune response would predispose to eumycetoma disease. This study compared eumycetoma patients with matched endemic controls without eumycetoma for co-infections with *Schistosoma* spp.. As a control, *Toxoplasma gondii* infections were monitored since this infection is also endemic in Sudan and results in a Th1-type of immune response in immune-competent hosts [17].

Although we only studied a limited number of people, which might not represent the full population of the study area, we did find an overall sero-prevalence of schistosomiasis in our study population of 51%, which is consistent with the earlier reported variable sero-prevalences for schistosomiasis in Sudan in the New Halfa and Um Zukra villages in the Gezira and Kassala regions (Figure 1) (16% and 70%, respectively) [27]–[29]. Large variations in prevalence of schistosomiasis even occur among villages in close proximity and depend on multiple factors, such as the environmental conditions for the intermediate snail host and the hygiene and bathing habits of the inhabitants [22]. The overall sero-prevalence of *Toxoplasma gondii* in our study population was 42%, which was exactly the same as found by Abdel-Hameed et al. in Gezira in 1991 [30]. This study now showed that eumycetoma patients were significantly more often sero-positive for schistosomiasis when compared to matched, endemic controls. The correlation is strengthened by the fact that antibody levels against *Toxoplasma*, another prevalent infection in Sudan, did not correlate with eumycetoma disease. Furthermore no correlation was found between seropositivity of schistosomiasis and toxoplasmosis.

The main underlying cause may be the strong Th2-response induced by schistosomiasis and the high expression of interleukin-10 (IL-10) by Th2 cells [16], [31]. IL-10 is capable of inhibiting synthesis of pro-inflammatory cytokines. Another anti-inflammatory trait of IL-10 is its potent ability to suppress the antigen-presentation capacity of antigen presenting cells. Increased IL-10 cytokine levels have also been detected in mycetoma lesions [7], [32] as well as in animal models of actinomycetoma caused by *Nocardia brasiliensis* [33]. Moreover, IL-10 levels were significantly elevated in serum of *M. mycetomatis* eumycetoma patients in Sudan [7], suggesting that IL-10 concentrations also play an important role in development or maintenance of eumycetoma.

In conclusion, even among this relatively small number of patients, eumycetoma was significantly associated with schistosomiasis and not with toxoplasmosis. Since this correlation is only based on serological data, animal studies are currently performed to investigate the precise role of schistosomiasis and Th2-predisposition in the development of fungal eumycetoma.

## Supporting Information

Checklist S1 STROBE checklist.
(DOC)
